# CBFA2T3::GLIS2融合基因阳性急性巨核细胞白血病3例报告并文献复习

**DOI:** 10.3760/cma.j.cn121090-20250814-00384

**Published:** 2026-03

**Authors:** 硕 林, 晔 郭, 丽 张, 玉梅 陈, 继刚 肖, 小矜 蔡, 文钰 杨, 晓娟 陈

**Affiliations:** 1 中国医学科学院血液病医院（中国医学科学院血液学研究所）、血液与健康全国重点实验室、国家血液系统疾病临床医学研究中心、细胞生态海河实验室，天津 300020 Institute of Hematology & Blood Diseases Hospital, Chinese Academy of Medical Sciences & Peking Union Medical College, State Key Laboratory of Experimental Hematology, National Clinical Research Center for Blood Diseases, Haihe Laboratory of Cell Ecosystem, Tianjin 300020, China; 2 天津医学健康研究院，天津 301600 Tianjin Institutes of Health Science, Tianjin 301600, China

## Abstract

回顾性分析中国医学科学院血液病医院收治的3例CBFA2T3::GLIS2融合基因阳性急性巨核细胞白血病（AMKL）患儿临床表现、遗传学特征、治疗经过及预后，并结合国内外文献对该罕见类型AMKL的诊疗特点进行总结。3例患儿中男1例，女2例，中位发病年龄16（8～19）个月，初诊时2例合并中枢神经系统白血病。FAB分型均为急性髓系白血病（AML）-M_7_，免疫表型均表现为CD56阳性、HLA-DR阴性，染色体核型均为复杂异常核型，其中2例涉及21号染色体三体。全部患儿经化疗均达形态学完全缓解，但均于早期复发，中位复发时间11（7～13）个月，中位总生存期16（15～47）个月。1例患儿首次移植后复发，二次移植后再次复发，现行姑息治疗；另2例复发后放弃治疗，最终死亡。结果显示，CBFA2T3::GLIS2融合基因阳性AMKL是一类侵袭性极强的亚型，常伴CD56阳性、HLA-DR阴性免疫表型及复杂核型，部分病例涉及21号染色体三体。该类患儿复发率高，预后极差。目前尚缺乏有效的治疗手段，亟需探索新的治疗策略以期改善结局。

## Abstract (en)

This study retrospectively analyzed the clinical manifestations, genetic characteristics, treatment courses, and prognoses of patients with pediatric acute megakaryoblastic leukemia（AMKL）with CBFA2T3::GLIS2 fusion treated at the Institute of Hematology, Chinese Academy of Medical Sciences. Further, the diagnostic and therapeutic features of this rare AMKL subtype, in conjunction with domestic and international literature, were summarized. The patients comprised one male and two females, with a median age at onset of 16 months（range, 8–19 months）. Central nervous system leukemia was present at diagnosis in two cases. All were classified as acute myeloid leukemia（AML）-M_7_ according to the French-American-British classification, with immunophenotypes demonstrating CD56 positivity and human leukocyte antigen-DR negativity. All patients showed complex abnormal karyotypes, including trisomy 21 in two cases. All patients achieved morphological complete remission（CR）after chemotherapy. However, all patients experienced early relapse, with a median relapse time of 11 months（range, 7–13 months）, and the median overall survival time was 16 months（range, 15–47 months）. One patient relapsed after the first hematopoietic stem cell transplantation and relapsed again after a second transplantation and is currently receiving palliative care. The other two patients discontinued treatment after relapse and subsequently died. These results reveal that CBFA2T3::GLIS2 fusion-positive AMKL is a highly aggressive subtype, frequently associated with a CD56-positive, HLA-DR–negative immunophenotype, and complex karyotypes, with trisomy 21 observed in a subset of cases. This subtype is characterized by a high relapse rate and an extremely poor prognosis. At present, effective therapeutic approaches are lacking, with an urgent need to explore novel treatment strategies to improve clinical outcomes.

CBFA2T3::GLIS2融合基因阳性急性巨核细胞白血病（AMKL）是一种罕见的急性髓系白血病（AML）亚型，主要见于非唐氏综合征相关AMKL（non-DS-AMKL）患儿，该融合基因亦可发生于部分细胞遗传学正常的儿童AML中，发生率约8％[1]。该亚型患者多起病于婴幼儿期，具有特征性免疫表型，病程进展迅速，整体预后较差[2]。传统化疗方案疗效有限，复发率高，无事件生存（EFS）率仅为8％～33％[3]。依据2022年WHO第5版造血与淋巴组织肿瘤分类，该类型AML被归类为“具有其他明确遗传学改变的AML亚型”[4]。根据我国儿童AML诊疗专家共识[5]及国际指南[6]，CBFA2T3::GLIS2融合基因阳性AML被归为高危组进行危险分层管理。国内针对该亚型的研究报道较少。本文回顾性分析中国医学科学院血液病医院收治的3例CBFA2T3::GLIS2融合基因阳性AMKL患儿的临床和实验室资料，并结合文献复习，旨在加深对该罕见AML亚型的认识，提升临床诊疗水平。

## 病例资料

例1，女，8月龄，因“发热8 d，伴血象异常6 d”于2021年8月16日就诊，入院血常规：WBC 258.3×10^9^/L，HGB 60 g/L，PLT 34×10^9^/L。骨髓象：增生活跃，异常原幼巨核细胞占65.2％，粒系及红系增生低下，巨核细胞未见，血小板散在可见，考虑AML-M_7_；免疫分型（静脉血）：异常髓系原始细胞占有核细胞的77.2％，表达CD56、CD33、CD34、CD117、CD13、CD38，不表达HLA-DR、MPO等（外周血涂片见图1A）；免疫组织化学染色：CD41阳性率74％。染色体核型为47,XX,del（16）（q22）,+21[8]/46,XX[2]；CBFA2T3::GLIS2融合基因阳性，WT1阳性。头颅CT示双侧大脑半球多发结节样稍高密度影，脑脊液流式细胞术分析可见异常髓系原始细胞，诊断中枢神经系统白血病（CNSL）。患儿经含克拉屈滨的联合方案化疗2个疗程达形态学完全缓解（CR），但融合基因持续阳性。CR后序贯2个疗程化疗，骨髓微小残留病（MRD）为6.00％，行脐带血造血干细胞移植（HSCT）。移植后第7个月患儿再次复发，检出IDH1、SETBP1新发突变，随后接受父供单倍体外周血HSCT，二次移植后第23个月再次复发，检出BRCA2、PALB2、DKC1等突变。多轮挽救治疗效果不佳。

**图1:**
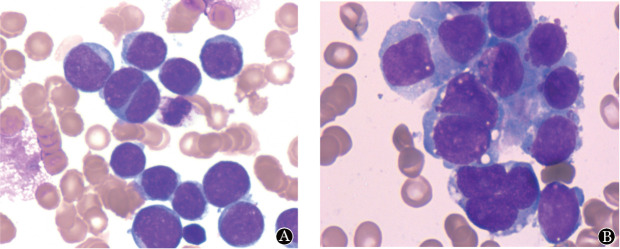
CBFA2T3::GLIS2融合基因阳性急性巨核细胞白血病患者的细胞形态学（瑞氏-吉姆萨染色，×1 000） **A** 例1外周血涂片；**B** 例2骨髓细胞形态学涂片

例2，女，16月龄，因“发热15 d余，血小板减少2周”于2021年5月25日就诊外院，血常规示WBC 16.7×10^9^/L，HGB 103 g/L，PLT 67×10^9^/L。外院确诊AML伴CBFA2T3::GLIS2融合基因阳性，脑脊液流式细胞术检测未见异常。外院行伊达比星联合阿糖胞苷诱导化疗1个疗程后达CR，随后行6个疗程巩固治疗，后复发转入我院。入院血常规WBC 3.81×10^9^/L，HGB 135 g/L，PLT 47×10⁹/L；骨髓原始细胞占45.5％，流式细胞术免疫分型：异常细胞群占44.57％，强表达CD56、CD117、CD33，部分表达CD34，弱表达CD123、CD38、CD13，不表达HLA-DR、CD45、TdT、cCD79a、MPO等髓、淋系标志物（骨髓形态见图1B，流式细胞术免疫表型见图2）。骨髓活检示骨髓增生活跃，幼稚细胞弥漫增多，粒红系细胞散在分布，巨核细胞少，MF-0级。免疫组织化学染色：CD41阳性率30％。染色体核型为46,XX,t（1;5）（q32;p15.3）[6]/46,XX[20]；WT1阳性。CBFA2T3::GLIS2定量309.2％，并检出CUX1、CTCF突变。患儿诊断AML-M_7_复发，予西达本胺联合化疗后骨髓未缓解，家属放弃治疗。

**图2:**
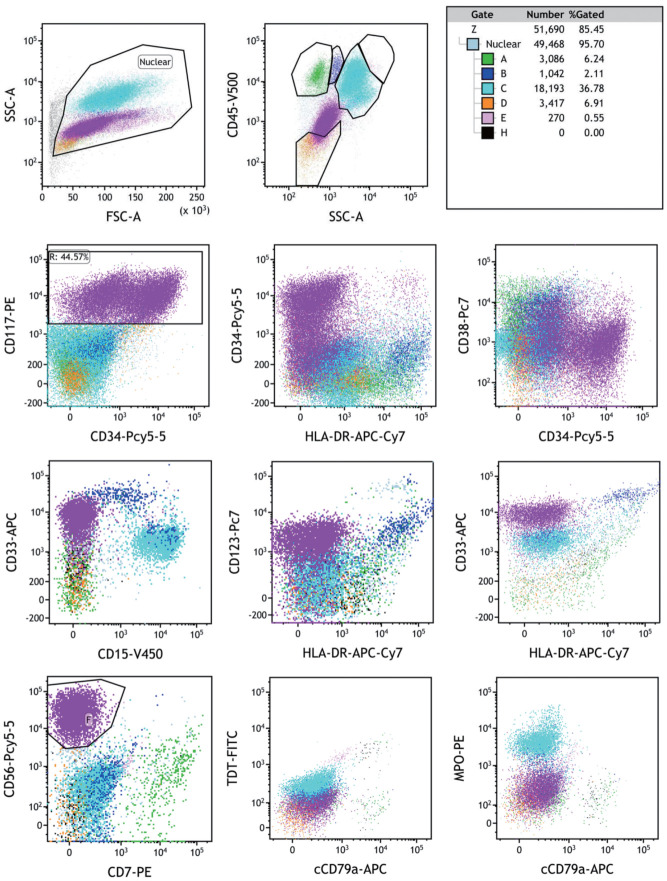
CBFA2T3::GLIS2融合基因阳性急性巨核细胞白血病患者（例2）骨髓流式细胞术肿瘤细胞免疫表型 **注** 异常细胞群占有核细胞的44.57％，强表达CD56、CD117、CD33，部分表达CD34，弱表达CD123、CD38、CD13，不表达HLA-DR、CD45、TdT、cCD79a、MPO等髓、淋系标志物

例3，男，19月龄，因“发热10 d”于2020年2月18日就诊，入院血常规WBC 26.8×10^9^/L，HGB 72 g/L，PLT 524×10^9^/L；骨髓象示增生活跃，原始细胞占52.0％，大小形态不规则，染色质较粗糙，核型不规则，核仁不明显，有核出芽状，胞质量丰富，多见伪足突起，POX染色阴性。流式细胞术免疫分型：异常细胞占有核细胞的12.8％，强表达CD56，表达CD33、CD81、CD117，不表达HLA-DR、MPO等。经脑脊液检查诊断CNSL。染色体核型：47,XY,+3[11]/48,idem,+21[2]/46,XY[7]，患儿诊断为AML伴CNSL。接受柔红霉素、阿糖胞苷及高三尖杉酯碱诱导化疗1个疗程后达CR，检出MPL、GNA13突变；CR后予4个疗程巩固化疗，诊断后约13个月复查骨髓MRD为40.73％，免疫组织化学染色CD41阳性率33％。患儿复发后一般状态差，家属放弃治疗，3例患者临床特征及预后见表1。

**表1:** 3例CBFA2T3::GLIS2融合基因阳性急性巨核细胞白血病患者临床特征及预后

例号	性别	年龄 （月）	WBC （×10^9^/L）	HGB （g/L）	PLT （×10^9^/L）	骨髓原始细胞（％）	FAB分型	染色体核型	CNSL （是/否）	复发 （是/否）	移植 （是/否）	生存情况	RFS期 （月）	OS期 （月）
1	女	8	258.3	60	34	65.2	M_7_	47,XX,del（16）（q22）,+21[8]/46,XX[2]	是	是	是	生存	7	47
2	女	16	16.7	103	67	45.5^a^	M_7_	46,XX,t（1;5）（q32;p15.3）[6]/46,XX[20]	否	是	否	死亡	11	15
3	男	19	26.8	72	524	52.0	M_7_	47,XY,+3[11]/48,idem,+21[2]/46,XY[7]	是	是	否	死亡	13	16

**注** WBC：白细胞计数；HGB：血红蛋白；PLT：血小板计数；CNSL：中枢神经系统白血病；RFS：无复发生存；OS：总生存。^a^患儿复发后骨髓原始细胞比例

## 讨论及文献复习

AMKL是AML的一类亚型，FAB分型为AML-M_7_[5]，约占儿童AML患儿的7.8％[7]。依据是否伴有唐氏综合征（Down syndrome，DS），AMKL可分为DS-AMKL和non-DS-AMKL，后者预后较差[8]。CBFA2T3::GLIS2融合基因是non-DS-AMKL中最常见且极具侵袭性的融合基因，发生率约为18.4％[9]，尤其多见于3岁以下婴幼儿AML患者[10]。此类患儿预后极差，5年生存率不足30％[3]。

CBFA2T3（又称MTG16/ETO2）基因位于16q24.3，作为转录辅抑制因子[11]，参与调控红系-巨核细胞分化[12]，促进白血病干细胞扩增与AML复发[13]。GLIS2基因位于16p13.3，与GLI家族相关[14]，在正常分化造血细胞中不表达，与CBFA2T3融合后异常激活[15]。二者通过inv（16）（p13.3q24.3）形成融合基因，保留CBFA2T3的NHR1-3结构域与GLIS2的ZF1-5及TRD结构[16]。该融合基因异常激活SHH、WNT及BMP等信号通路，上调ERG、KIT基因表达[15]–[17]，增强造血干细胞的自我更新，同时抑制GATA1表达[18]，阻断细胞分化，致原始细胞积聚，发挥致白血病作用[19]。此外，其显著富集于CD56启动子区，驱动其高表达[20]。

迄今为止，CBFA2T3::GLIS2融合基因仅见于儿童AML，Masetti等[1]研究表明其并不局限于FAB-M_7_，亦可存在于其他FAB亚型中。该类患儿临床表现缺乏特异性，骨髓中原始巨核细胞呈多形性、成簇分布，胞质可见空泡或伪足[21]。CBFA2T3::GLIS2融合基因由隐匿性inv（16）形成，常伴21号染色体三体或复杂核型[9],[22]，常规核型分析难以检出，诊断存在一定挑战。近年来RNA测序Panel技术提高其检出率，有助于早期诊断。此类患者免疫表型常与“RAM表型”相关[23]，呈CD56高表达，伴HLA-DR阴性表达等，其中CD56被认为是最具诊断价值的免疫标志物[3]。本研究3例患儿均以发热起病，女性2例，男性1例，发病年龄均小于2岁，其中2例患儿合并CNSL。患儿免疫表型均表现为CD56阳性，HLA-DR阴性，符合RAM表型特征。染色体核型具有显著异质性，正常与异常核型共存，与既往报道一致[9],[22]。其中2例患儿伴21号染色体三体，1例同时合并3号染色体三体，此前亦有病例报道[24]，其致病机制及预后意义仍有待进一步探索。此外，除融合基因外，患儿均检测到多种复杂基因突变，提示其发病可能涉及多种分子机制协同作用。复发时常伴新的基因突变产生，可能与疾病克隆演化、耐药性产生及病情进展相关。

CBFA2T3::GLIS2融合基因阳性AML是高度侵袭性、预后极差的罕见分子亚型。Gruber等[16]于2012年首次在儿童AMKL中报道该融合基因，将其归为预后不良亚组，5年OS率仅8％～33％[3]。de Rooij等[2]在153例AMKL患儿的研究中发现，CBFA2T3::GLIS2为最常见的融合基因，阳性率16％，阳性患儿4年EFS率仅为33％。Smith等[25]报道儿童肿瘤协作组AML队列中CBFA2T3::GLIS2阳性率为1.9％，患者中位年龄为1.5岁，与诱导治疗失败、复发及死亡密切相关，5年EFS率仅为18.9％。本研究中的3例患儿经化疗后均可实现骨髓形态学CR，但融合基因或MRD持续阳性，缓解状态难以长期维持。3例患儿均早期复发，中位复发时间11个月，中位生存时间为16个月。复发后病情进展迅速，治疗反应欠佳，截至末次随访2例患儿复发后放弃治疗死亡，1例姑息治疗。

针对该复发难治性AML亚型，现有常规化疗及靶向治疗疗效有限，异基因HSCT仍是目前主要根治手段。Du等[26]报道6例CBFA2T3::GLIS2融合基因阳性AML患儿中，4例接受单倍体相合HSCT治疗后生存良好，证实HSCT可改善患儿预后。本研究1例接受HSCT治疗，延长患儿生存期，但移植后病情反复，考虑和移植前MRD>5％，疾病负荷较高相关。提示在CR1期、MRD阴性状态下行HSCT可能获益，尚需更大样本、前瞻性研究验证。目前，针对CBFA2T3::GLIS2融合基因阳性AML的精准治疗正在探索。针对该融合基因形成机制及其致白血病作用，GLI抑制剂GANT61可显著抑制该亚型细胞增殖并诱导细胞凋亡[20]。基于特征性CD56高表达，抗CD56抗体药物偶联物对患者来源细胞表现出显著毒性作用，有望成为新靶向治疗策略[25]。Le等[27]鉴定出叶酸受体（FOLR1）为CBFA2T3::GLIS2融合基因阳性AMKL的潜在治疗靶点，并验证了FOLR1-CAR-T细胞的杀伤活性，提供新的治疗思路。

综上所述，CBFA2T3::GLIS2融合基因阳性AMKL是高度侵袭性的AML亚型，好发于婴幼儿，具有特征性RAM免疫表型和复杂染色体核型异常，常伴有中枢神经系统浸润。高通量测序技术提高该类携带罕见融合基因患儿的检出率。尽管部分患儿诱导化疗后可达形态学缓解，但早期复发率高、复发后救治困难，导致总体预后不良。现有治疗手段有限，未来亟需探索更有效的靶向治疗及联合策略，以期改善患儿远期生存。
